# An auditory Charles Bonnet Syndrome managed with psychological intervention: A case report

**DOI:** 10.1192/j.eurpsy.2023.1604

**Published:** 2023-07-19

**Authors:** M. Karoui, A. Mediouni, H. Nefzi, F. Ellouze

**Affiliations:** 1 Psychiatrie, Faculté de médecine de Tunis, Tunis, Tunisia; 2 ORL, Faculté de médecine de Tunis, Tunis; 3 Psychiatrie, Faculté de médecine de Tunis, Tunis

## Abstract

**Introduction:**

Charles Bonnet Syndrome (CBS) is an age-related disorder characterized by complex visual hallucinations in older persons with vision loss and underlying ocular pathology. The management of these symptoms is imprecise and combines psychological measures with psychotropic drugs.

**Objectives:**

to discuss the non-pharmacological management of Bonnet syndrome through a case report.

**Methods:**

We report a case of atypical CBS in a 76-year-old male patient presenting with visual and auditory hallucinations that were improved by reassurance.

**Results:**

The past medical history was significant for diabetic retinopathy, difficulty hearing due to bilateral sensorineural hearing loss. He recognized these visions as unreal and felt distressed by them. No cognitive impairment was observed on several neuropsychological tests. He was reassured of the false nature of the visual experiences after explanations that he had no mental illness and that the problem could disappear. He was taught how to keep the images away by closing his eyes for sometimes and repeated blinking. After six weeks of psychological intervention, the visual experiences had disappeared without using any drug

**Conclusions:**

In the management of CBS drug treatments remain partially satisfactory. Nonpharmacological interventions focus on the reduction of the visual pathway deprivation. This therapeutic alternative seems to provide positive benefits.

**Disclosure of Interest:**

None Declared

